# Nurses’ Practices in the Peripheral Intravenous Catheterization of Adult Oncology Patients: A Mix-Method Study

**DOI:** 10.3390/jpm12020151

**Published:** 2022-01-24

**Authors:** Paulo Santos-Costa, Filipe Paiva-Santos, Liliana B. Sousa, Rafael A. Bernardes, Filipa Ventura, William David Fearnley, Anabela Salgueiro-Oliveira, Pedro Parreira, Margarida Vieira, João Graveto

**Affiliations:** 1 The Health Sciences Research Unit: Nursing, Nursing School of Coimbra, 3004-011 Coimbra, Portugal; filipesantos@esenfc.pt (F.P.-S.); baptliliana@esenfc.pt (L.B.S.); rafaelalvesbernardes@esenfc.pt (R.A.B.); filipaventura@esenfc.pt (F.V.); anabela@esenfc.pt (A.S.-O.); parreira@esenfc.pt (P.P.); jgraveto@esenfc.pt (J.G.); 2 Instituto Ciências da Saúde, Universidade Católica Portuguesa, 4169-005 Porto, Portugal; mmvieira@porto.ucp.pt; 3 King’s College Hospital NHS Foundation Trust, London SE5 9RS, UK; willfearnley1995@gmail.com; 4 Centre for Interdisciplinary Research in Health, Universidade Católica Portuguesa, 4169-005 Porto, Portugal

**Keywords:** oncology patients, peripheral intravenous catheterization, nurses, mix-method study

## Abstract

A significant number of adult oncology patients require at least one peripheral intravenous catheter to fulfill their therapeutic plan. Recent evidence indicates that catheter failure rates are high in this cohort, impacting care outcomes and patient experience during cancer treatment. This reality represents a challenge to nurses worldwide since in most international settings they are responsible for delivering quality care during the insertion and maintenance of such devices. This study aims to explore current nursing practices regarding the insertion, maintenance, and surveillance of peripheral intravenous catheters in oncology patients. A two-phase mix-method study was conducted with the nursing team from the surgical ward of a large oncology hospital in Portugal. In phase one (observational prospective study), nurses’ practices during catheter insertion and maintenance were observed by the research team and recorded using standardized instruments and validated scales. In phase two, three online focus groups were conducted with the nursing team to present the results observed in phase one and explore their perceptions of current practices. All ethical principles were assured throughout the study. Significant divergent practices were observed and identified by the nurses, especially concerning patient involvement, nurses’ adherence to the aseptic, non-touch technique, catheter stabilization and dressing, and catheter flushing and locking. Such practices may partially explain the high complication rate found (26%) and substantiate the need for future intervention in this field.

## 1. Introduction

The use of peripheral intravenous catheters is widespread in adult oncology patients, who receive different courses of intravenous treatment over a number of years [1]. Adult oncology patients often require long cycles of antineoplastic treatment, leading to endothelial dysfunction, loss of vasorelaxant effects, as well as suppressed anti-inflammatory and vascular reparative functions [1,2,3].

As the procedure of peripheral intravenous catheterization (PIVC) is invasive, there is a need for greater consistency during catheter insertion, management, and continuous surveillance. Complications related to PIVC, such as phlebitis, infiltration, and overall depletion of the peripheral venous network, increase significantly in oncology patients, with incidence rates varying between 34.9% and 50% [2,4,5,6].

This represents a significant challenge for nurses during the insertion, maintenance, and surveillance of peripheral intravenous catheters throughout most international settings [4,7]. However, identifying nursing-sensitive outcomes is not a linear process [7] since the results of nursing care are also dependent on patient and clinical setting-related variables.

Recent studies have addressed PIVC-related practices and outcomes in adult oncology patients, focusing on the identification of inherent and modifiable risk factors for PIVC difficulty and failure, mostly through single-day data collection methods or via secondary analysis [5,8,9,10,11]. Other authors have explored nurses’ knowledge, confidence levels, and adherence to local policies in this field [6,12,13,14].

To the best of our knowledge, there is a gap in the literature pertaining to both the continuous in loco observation of nurses’ practices during the insertion and maintenance of peripheral intravenous catheters in oncology patients, and the perceptions amongst nurses’ regarding current practices. Thus, this study aims to explore current nursing practices regarding the insertion, maintenance, and surveillance of peripheral intravenous catheters in oncology patients. As a secondary goal, we aim to explore nurses’ perceptions of current PIVC-related practices and identify areas that may require future intervention.

## 2. Materials and Methods

### 2.1. Study Design

A mix-method study was conducted in two phases: (i) observational prospective study in the selected study setting; (ii) focus groups rounds with the ward nurses.

### 2.2. Study Setting

In phase one, from December 2019 and July 2020, an observational prospective study was conducted in a surgical ward from an oncology hospital in Portugal. The ward’s nursing team (*n* = 28) was presented with the description and objectives of the study by the lead researcher. All ward nurses accepted to participate in the study.

The nursing team was mainly composed of female nurses (78.6%), with a median age of 39.5 years (24–58). The nursing team had a median of 16 (3–35) years of nursing experience, of which 15.5 (0–32) years were in the surgical ward. The vast majority (64.3%) held a nursing degree, although some possessed other academic titles, such as a bachelor’s degree (10.7%), post-graduate (3.6%), master’s degree (17.9%), and doctorate (3.6%). Six nurses had a specialization, specifically in medical-surgical nursing (*n* = 3), mental health nursing (*n* = 2), and pediatric nursing (*n* = 1).

In phase two, three online focus group rounds were conducted with the ward nurses with a double intent: (i) present the results of phase one and (ii) explore their perceptions on current PIVC-related practices. The online focus group rounds were conducted using the Microsoft Teams software and scheduled by ward manager.

### 2.3. Participants

Patient recruitment followed a non-probability, consecutive, and based on convenience sampling technique, indicated by the ward’s nursing team at the time of catheter insertion or maintenance. Patients that were selected had to be over 18 years of age, able to provide informed consent, and planned to return to the same ward after surgery. Patients with peripheral venous system damage, known intravenous drug addiction, and patients that would be transferred to another unit after the planned surgical procedure were excluded from the study.

### 2.4. Variables and Data Collection

In phase one, data were collected using a checklist developed by the research team, based on recent international standards of care and recommendations for a dataset in this field [15,16]. During the morning period (9 a.m. and 1 p.m.), nurses were accompanied by the lead researcher, who observed their practices during catheter insertion or maintenance. When necessary, the lead researcher would request further oral clarification to characterize nurses’ PIVC-related practices. After each observation, the lead researcher would thank the nurse and patient for their collaboration, move to another private room in the ward and fill the checklist. A single person collected data throughout the study to avoid chances of bias and missing data [17].

Variables recorded focused on patients’ demographic and clinical status, catheter insertion-related outcomes (e.g., selected puncture site, catheter gauge size, number of puncture attempts until success, catheter fixation method, antiseptic used, and nurse-reported ease of puncture through a Likert Scale ranging from 1 “not at all difficult” and 7 “extremely difficult” points), and catheter maintenance-related outcomes (e.g., indwell time, dressing integrity, and reason for premature removal).

Two culturally adapted scales were used to characterize patients’ peripheral intravenous network, the Escala Nacional de Acessos Vasculares (ENAV) and the Escala A-DIVA Modificada (A-DM) [18,19]. The ENAV is a “performance status tool” that prompts healthcare professionals to categorize a patient’s peripheral intravenous access according to three domains: (i) the number of observable puncture points; (ii) peripheral intravenous catheter size and ease of performing venipuncture; and (iii) risk of extravasation or phlebitis. The risk of difficult intravenous access progressively increases with each grade [19]. Contrastingly, the A-DM scale bases its assessment on five items: (i) the patient’s history of difficult intravenous access; (ii) expected difficult intravenous access by the healthcare professional before an attempt at catheter insertion; (iii–iv) the inability to detect a dilated vein by palpation and/or visualization; and (v) the diameter of the target vein being less than 3 mm. Each confirmed item adds one point to the scale’s score (ranging from 0 to 5), where a higher score indicates a higher risk of difficult intravenous access and risk of failed catheter insertion [18].

If the catheter was prematurely removed due to a complication, the signs and symptoms reported by the ward nurses were retrieved for analysis and subsequent classification as phlebitis, infiltration, obstruction, or accidental removal. To assist with such assessment, nurses were provided with the culturally adapted and validated versions of the Phlebitis Scale and Infiltration Scale [20,21]. The Phlebitis Scale is an instrument scored from zero (absence of phlebitis) to four (evident signs of thrombophlebitis), each level containing the signs and/or symptoms of phlebitis and assisting professionals to determine the need to replace the peripheral intravenous catheter [20]. The Portuguese version has two levels of severity with a Cronbach’s alpha of 0.78 (level one) and 0.90 (level two). The Infiltration Scale is organized into four levels, between zero (absence of infiltration) and four (severe infiltration), using clinical criteria to evaluate each level of infiltration, such as skin colour, skin temperature to touch, pain, extent, and depth of oedema [21]. The Portuguese version has a Cronbach’s alpha of 0.85.

In phase two, the data collection period was October to November 2020. Three focus groups were conducted to involve as many elements of the nursing team as possible. The focus groups were conducted online, through Microsoft Teams software, and lasted for 70–96 min. The focus groups were audio-recorded with participants’ written consent, transcribed verbatim and findings are supported by direct quotations to allow the reader to judge the dependability. Data were de-identified using pseudonyms. Before presenting the results of the observational study, the research team presented six statements concerning the team’s PIVC-related practices and asked each nurse to rate their accordance with each statement anonymously using a 1 (completely disagree) to 10 (completely agree) Likert Scale. This initial questionnaire was applied using an online software tool (www.menti.com, accessed on 1 October 2021), and included the questions “During catheter insertion, the team adheres to hand hygiene measures”, “The person is involved before, during, and after catheter insertion”, “Insertion success rate is high”, “Aseptic technique principles are followed during catheter insertion”, “Current catheter maintenance care practices (e.g., assessing dressing viability, catheter flushing/locking) follow international recommendations”, “The current PIVC-related complication rate is low”.

### 2.5. Ethics

Before study commencement, nurses’ voluntary and informed consent to participate was obtained by the lead researcher, who assured that all ethical assumptions in clinical research would be complied with. All included patients were also informed of the ongoing study by the lead researcher, who ensured their voluntary and informed consent to participation. This study was approved by the hospital’s board after a favourable review by its Ethics Committee (ref. TI 24/2019, approved on 19 September 2019).

### 2.6. Data Analysis

Quantitative data analysis was performed using SPSS Statistics^®^ (version 24, IBM SPSS; Chicago, IL, USA), using means, standard deviations, frequencies, and percentages as descriptive statistics. Concerning the qualitative data retrieved in phase two, recordings were transcribed verbatim, anonymized, and uploaded to the QSR Nvivo10 (QSR International, Melbourne, Australia) qualitative software programme. Two members of the research team performed thematic content analysis following the Bardin technique [22]. The emerging categories and subcategories were discussed among the researchers to develop their structure.

## 3. Results

During phase one, 100 patients were enrolled in the study (Table 1), 83% of whom required a peripheral intravenous catheter due to an impending surgery, while 12% had a previous non-functioning catheter.

### 3.1. Phase 1—Observation of Current PIVC-Related Practices

#### 3.1.1. Peripheral Intravenous Catheter Insertion

Most of the nurses (95%) asked for the patient’s informed consent before catheter insertion. Nurses checked the patient’s peripheral veins starting from the back of the hand up to the antecubital fossa (96%). However, just 25% of the nurses asked about the patient’s previous peripheral catheterization experiences. No assessment instrument was routinely used by the nursing team to assess the potential level of peripheral vascular access difficulty.

After finding a suitable vein, nurses adjusted the disposition of the patient’s surroundings (96%), mostly due to the need to sit down next to the selected arm (89%) or to increase vein visibility due to poor lighting (27%).

Generally, all nurses confirmed the patient’s identity once more before starting the procedure, although verbally with the confirmation of the patient’s name. Before commencing the procedure, 90% of the nurses instructed the person on what to do during the puncture to attempt to control pain and avoid sudden movements of the upper limb.

Most of the nurses (86%) selected an optimal puncture site, locating a vein in the dorsal and ventral surfaces of the upper extremities, including the metacarpal (59%) and cephalic, median or basilic veins (34%). No observations were made of puncture attempts in bony areas, areas near infected, inflamed, or broken skin, as well as flexion areas. All observed catheter insertions were performed in an upper extremity.

In 96% of the observations, textile reusable tourniquets were applied without a previous attempt to clean/disinfect them by spraying alcohol or clorohexidine. In two observations, nurses used a rubber tourniquet without previous decontamination, while two nurses used clean gloves as a tourniquet. Regardless of the type of tourniquet used, all nurses applied it 5–10 cm above the desired puncture site. Nonetheless, only 12% relieved the tourniquet after finding a potential puncture site and before preparing the remaining material. To enhance vein distension, 47% of the nurses employed other strategies such as instructing the patient to open and close their hand repeatedly, closing their hand intensely (“making a fist”), rubbing their hands or performing gentle taps on the selected puncture site location.

In 79% of the observations, the selected peripheral intravenous catheter was a 20-gauge, followed by the 22-gauge (14%). After vein selection, nurses wore gloves in 23% of the observed catheterizations. For site antisepsis, the majority preferred 2% chlorhexidine gluconate in 70% isopropyl alcohol (83%), followed by 70% isopropyl alcohol (16%). However, most nurses (72%) did not allow for the antiseptic to completely dry off before attempting catheter insertion. Moreover, 51% of the nurses manipulated the insertion site after applying the antiseptic and did not perform skin antisepsis once more.

All nurses used a safety-engineered catheter, with double flashback technology and an anti-reflux valve. Before attempting catheter insertion, 61% of the nurses warned the patient that they would feel a skin prick. All catheter insertions were performed with an initial angle of 10°–30°. Upon visualizing blood reflux, all nurses decrease the catheter angle and progressed the plastic tube while retracting the stylet. Peripheral intravenous catheter insertion was successful on the first attempt in 67% of the moments, with an average of 1.57 puncture attempts (1–8, SD ± 1.1). In total, in most of the observed catheterizations (92%), nurses closed the system by attaching a needleless connector to the catheter, while in the remaining situations nurses attached a giving set directly to the catheter.

All the inserted peripheral intravenous catheters were fixated with a transparent film dressing, although nine catheter insertion sites were covered with adhesive strips. Only four dressings were identified with the insertion date. Post-insertion flushing was performed 67% of the time, although with high variability in volume and technique. All nurses selected 0.9% preservative-free sodium chloride to perform catheter flush, although volumes varied between 2 mL (*n* = 12), 3 mL (*n* = 39), and 5–10 mL (*n* = 16). The use of the push-pause technique was not observed during the study, with nurses performing catheter flush as a single bolus injection.

After the procedure, 54% of the nurses questioned the patient about his/her comfort, and 19% informed the patient about potential complications, associated signs and symptoms, and preventive strategies during mealtimes, showering, and dressing/undressing gowns and robes.

Overall, during catheter insertion, nurses’ hand hygiene compliance rates were documented following international recommendations for procedure duration, solution, and technique. Results can be found in Table 2.

On average, nurses spent 11.8 min (3–50, SD ± 7.5) to successfully insert a peripheral intravenous catheter.

#### 3.1.2. Peripheral Intravenous Catheters Maintenance

Peripheral intravenous catheters remained in situ for 2.1 days (0–8, SD ± 1.4). Overall, most nurses (94%) adhere to the standard aseptic non-touch technique when accessing/changing the needless connector. Ninety-two per cent of the catheters had a luer-locking needle connector attached. Overall, in 54% of the observations made, nurses scrubbed the needless connector before each use, usually with alcohol-based chlorhexidine gluconate or 70% isopropyl alcohol.

Eighty-five per cent of the nurses assessed catheter function with a 0.9% sodium chloride flush before and after each use, although flushing volumes varied greatly with only 40% of the nurses using a 5–10 mL flushing volume. Catheter lock with a 0.9% preservative-free sodium chloride was performed 67% of the time. Moreover, no observations were made of nurses complying with the push-pause technique.

Fifty-two observations were made of nurses routinely assessing the insertion site and surrounding area to identify potential complications, although most of the nurses were aware of the catheter had been used in the previous 24 h (70%). After surgery, if no intravenous therapy was prescribed, all peripheral intravenous catheters remained in situ for 24 h.

Routine dressing changes and site care were performed in 52% of the observations. Dressing changes were mostly due to its integrity becoming compromised after the patient’s first post-surgical shower.

Nurses’ compliance rates with hand hygiene measures during catheter maintenance can be found in Table 3.

Throughout the study, a complication rate of 26% was recorded, mainly due to infiltration (18%) and phlebitis (9%).

### 3.2. Phase 2—Nurses’ Perceptions of Current Practices

Concerning the online questionnaire applied, statements were rated between 7.8 and 8.3 points, indicating a positive self-assessment of current practices. Content analysis revealed different subcategories, which were grouped into two main categories titled “challenges in PIVC care with oncology patients” and “nurses’ PIVC care practices”.

#### 3.2.1. Challenges in PIVC Care with Oncology Patients

A common struggle highlighted by nurses was the previous antineoplastic treatment that patients experienced before ward admission, which conditioned the peripheral intravenous network preservation.

“Oncology patients have specific characteristics when it comes to vein preservation” (Specialist nurse, female, 22 years of nursing)

“If a patient underwent chemotherapy, especially for breast cancer treatment, the peripheral venous network becomes almost invisible and difficult to cannulate (…) and it is something that we (nurses) cannot change… but we can implement strategies that minimize the impact of chemotherapy” (Specialist Nurse, male, 10 years of professional experience)

“My experience is similar (…) if I know that I have to cannulate a patient that underwent chemotherapy for breast cancer treatment, I will panic! The venous pattern disappears, and it becomes almost imperceptible… adding extra difficulties to catheter insertion” (Specialist nurse, male, 10 years of professional experience)

“(At our hospital) the fact that only a few patients undergo chemotherapy through central accesses is extremely hard for patients (…) they suffer during the cycles of chemo through peripheral veins, and continue to suffer during the preoperative period with the constant puncture attempts to insert a viable peripheral intravenous catheter” (Registered nurse, female, 25 years of professional experience)

“The number of chemotherapy cycles increases the difficulty in locating a suitable vein, as well as the venipuncture itself” (Registered nurse, female, 23 years of professional experience)

Nurses were aware that such challenges must be addressed by the nursing team, focusing on a thorough assessment of the patients’ history of difficult peripheral intravenous catheterization as well as current risk factors:

“We cannot change the profile of our patients, but we do need to improve our care practices, reducing their pain and stress levels during peripheral intravenous catheterization” (Specialist nurse, male, 10 years of professional experience)

“We need to study their peripheral venous pattern…and assess if veins are visible and palpable, if the patient is overweight, diabetic (…) we should be able to conduct such assessment “naturally” (Specialist nurse, female, 11 years of professional experience)

#### 3.2.2. Nurses’ PIVC Care Practices

Nurses identified a few strategies that were used to locate and select a suitable vein in patients that underwent antineoplastic treatment:

“(…) Using heated packages of saline or ask the patients to hold down their arms for a few minutes, which will hopefully allow for vein distension. Heated saline packages help a lot… it is a good strategy that has saved me a lot of times… but it is not a common practice (in the ward)” (Specialist nurse, male, 10 years of professional experience)

“I often use gauze impregnated with alcohol and massage the veins to distend them (…) but I also ask the patient to lower their arms for a few minutes” (Registered nurse, female, 34 years of professional experience)

Nurses’ flushing practices were also discussed, with some expressing concern with the current risk of infection associated with this procedure due to the lack of individualized resources and safe practices:

“I usually aspirate normal saline to a 5 mL syringe (…) from a single drip bag that is hanging in the medication room (…) everyone has access to the same bag, which is used for preparing catheter flushes and dilute intravenous drugs… the bag has an aspiration device attached, that after being disinfected, we can connect a normal syringe…” (Specialist nurse, female, 11 years of professional experience)

“How many of us disinfect the aspiration device before aspirating the normal saline? We hang a litre drip bag…because, you know… given our high admission and discharge rate, if we hang a 100 mL drip bag, it will be emptied quickly, right? We use a litre drip bag to facilitate our work a little bit more… but to what extent is this a viable practice? We have normal saline ampoules and know that it would be correct to use them for each patient…” (Specialist nurse, female, 11 years of professional experience)

“We have clinical material and devices of high quality… I believe there is an institutional effort to provide us with the best available resources in high quantities… [but] when we prepare the material needed for catheter insertion, we usually forget about the material needed to perform catheter flushing” (Registered nurse, female, 14 years of professional experience)

Another practice that was highly debated between nurses was glove usage during catheter insertion.

“I must confess that I rarely use gloves… because I have some difficulty in identifying a proper vein while wearing gloves. The risk of contracting with blood…well, our catheters have an antireflux valve…and thus the risk is minimized… I will only use gloves if I am aware that the person has an infectious disease” (Specialist nurse, male, 11 years of professional experience)

**“** (I do not use gloves) because using them decreases sensibility in the tip of the finger which makes our job harder when we are palpating a vein before attempting to puncture it” (Registered nurse, female, 22 years of professional experience)

Some nurses also identified the use of reusable tourniquets as a potentially unsafe practice that can pose a risk of cross-infection:

“Concerning our equipment and materials, I believe that our institution is concerned with providing us with high-quality material… but on several occasions, our use of tourniquets diverges from current standards of care. The fact that these devices are stored in different spaces of the treatment room can also make it harder for nurses to prepare the material needed for catheter insertion… we may even prepare the material that is required, but hardly put it back in the same spaces… this makes it harder for nurses to locate the required material in a short amount of time” (Registered nurse, female, 21 years of professional practice)

“Our tourniquets are not decontaminated between patients, and I believe this is not a correct practice, which influences the safety of the procedure” (Specialist nurse, female, 11 years of professional practice).

A debate was generated during the focus group concerning nurses’ current dressing care practices:

“I always write the insertion date in the dressing… because I have identified that some catheters remained in situ over 72 hours. Some colleagues change the date written every time they perform a dressing change…but the date that is written refers to the insertion date” (Registered nurse, female, 14 years of professional practice)

“I believe that our colleagues are increasingly writing the insertion date on the catheter dressing since we used to do it regularly in patients with totally implanted catheters (…) and we will achieve that with peripheral intravenous catheters as well” (Registered nurse, female, 17 years of professional practice)

“I do not write down the insertion date because when we perform dressing change, and we do it daily, that date is lost between changes” (Registered nurse, female, 13 years of professional practice)

“I only change the dressing to ensure that the insertion site is preserved… our dressings boarders will often become loose after the patient showers… the use of dressings with reinforced borders could be a solution to implement in our ward” (Specialist nurse, male, 10 years of professional practice)

“Unfortunately, I often see bad practices here in our ward, with some nurses placing adhesive strips over adhesive strips…” (Registered nurse, female, 14 years of professional practice)

The previous topic led the participants to discuss the lack of an agreement concerning what PIVC-related care interventions should be performed in a uniform way, as well as how to record them in the patients’ clinical notes:

“When we started writing our nursing notes on SAPE (a clinical computer software used in Portugal), we agreed as a team to include in the patients’ care plan the daily intervention “perform catheter maintenance care”…because if this intervention was included as an S.O.S., nobody would select it…this was decided 11 years ago” (Specialist nurse, female, 14 years of professional practice)

“There is also the intervention (on the SAPE software) “monitor inflammatory signs” that should be performed every shift” (Specialist nurse, Male, 10 years of professional practice)

“I believe that if the intervention “perform catheter maintenance care” is included in the S.O.S. list, nobody will perform it…” (Registered nurse, female, 23 years of professional practice)

“We are talking about good practices… it is not the fact that such interventions are included in the care plan that will make us remember whether or not to carry it out (Specialist nurse, female, 11 years of professional practice).

“The truth is…our nursing care documentation often fails to report what in reality is done… this is the only explanation… because we often see a nursing note made by a colleague that reports “no inflammatory signs”, and when I see the patient’s insertion site there is exuberant phlebitis, with palpable venous cord, oedema, redness… and we then understand that we could prevent this if it was one of our main concerns (…) Every intervention that does not require nurses to look and grade it with a scale…most of us will just click on the performed button, assume that the catheter is working, everything is normal…and we continue to find phlebitis in the upcoming shifts” (Specialist nurse, male, 10 years of professional practice)

“Some notes do not report what is done by nurses and what is happening… (I am sad) to see these episodes of phlebitis, that often happen due to bad practices performed by nurses who should be taking care of people in need…and then record interventions that are not performed…” (Specialist nurse, female, 35 years of professional practice)

## 4. Discussion

During the first phase of this study, the observed insertion and maintenance practices differed greatly between nurses and, at times, from well-accepted international standard of care recommendations. Although some results may be explained due to contextual factors, specific care practices must be discussed in detail to identify the associated deficits, challenges, and clinical outcomes for oncology patients [16].

One of these practices concerns the initial assessment of the patients’ venous network and past experiences in vascular access. In our study, during the focus groups (phase two), nurses spontaneously shared their catheter insertion experiences with oncology patients, reinforcing that this specific cohort poses a challenge to clinicians during vein location and selection [1,12]. Oncology patients are subjected to cycles of vesicant antineoplastic treatment and long periods of hyperosmolar intravenous therapy, both of which damage the peripheral veins and contribute to a loss of potential puncture sites. During phase one of this study, in a noticeable contradiction, patient involvement was observed in only a quarter of all observations, reducing opportunities to identify risk factors for difficulty in peripheral intravenous catheter insertion. We found that no instruments were used by nurses to standardize this initial approach, leading to the suboptimal assessment between nurses and poor documentation, affecting care continuity [23].

Failing to perform a comprehensive assessment of the patient’s vascular network can result in multiple failed puncture attempts and depletion of peripheral veins [18,19,24]. Likewise, overlooking patients’ past experiences can result in poor care outcomes, since some patients experience regular failing catheterization due to complications, such as phlebitis, infiltration, and extravasation, which can be preventable if diagnosed early [25]. According to the Infusion Nurses Society, nurses are expected to employ in their care process a “holistic, patient-centered approach to safely deliver infusion therapy and perform vascular access insertion and management” [16]. Such an approach must focus on preserving patients’ vessels, preventing complications, and maximizing the patient’s positive experience [16,25,26].

In the emerging era of precision medicine, the inclusion of person-centered elements into the comprehensive assessment of patients’ vascular network might successfully contribute to personalized care [27]. Facing the challenges in integrating precision medicine and technology in person-centered care [28], the results of this study particularly highlight the importance of attaining to everyone’s genotype, phenotype, and lifestyle elements along with the vascular assessment. As such, person-centered elements will most likely influence PIVC success and patient’s experience. Through translational research, the person-centered causes underlying successful and unsuccessful PIVC procedures might be uncovered and further guide best practices guidelines concerning PIVC and vascular network assessment in oncology patients [29].

Another area that requires further discussion is the use of reusable equipment between patients, especially tourniquets. As identified by the ward nurses in phase two, no current decontamination protocol is in place, resulting in the contamination of this device with potentially pathogenic microorganisms, such as *Staphylococcus* coagulase-negative and *Staphylococcus aureus*, with high resistance profiles [30]. Tourniquet contamination, especially of those found in this study setting which are made of fabric, can lead to direct (tourniquet is placed near the selected puncture site) or indirect cross-contamination (contaminating other material and nurses’ hands) [30,31]. Nurses working in oncology settings must be aware of such implications due to the potential compromise of the patients’ immune system due to previous cycles of antineoplastic treatment [32]. In Portugal, even after the COVID-19 outbreak, most clinical settings still use reusable tourniquets made of fabric. A recent study conducted in a cardiology ward showed that single-use disposable tourniquets decrease the risk of contamination of the puncture site and catheter lumen with pathogenic microorganisms, with potential long-term improvements in catheter-related bloodstream infection rates [33]. In this study, nurses reported that having quality equipment available in sufficient quantity is a concern of the ward/hospitals managers. Future studies must focus on the potential implementation of such innovative devices as a viable solution that enhances care quality and safety during catheter insertion in oncology settings.

Another area for improvement concerns nurses’ adherence to the antiseptic non-touch technique (ANTT), with several associated practices that diverge between nurses and do not comply with international standards of care. Nurses donned gloves in only 23% of the catheter insertion moments, lower than what is reported in several international studies focused on catheter insertion and maintenance [34]. During the focus groups rounds, nurses admitted that they rarely use this protective equipment due to a loss of tactile sensitivity that increases the already difficult task of finding a suitable vein. However, with most patients, gloves are not required during vein location and selection, which can be performed safely by nurses with prior hand hygiene [16,35]. During the focus groups, the lack of glove usage was also explained by the mechanics of the peripheral intravenous catheters in use at the ward, which accommodate double-flashback technology and passive safety systems, decreasing the risk of needlestick injury and contact with patients’ blood. However, such devices are not risk-free and should always be used following international standards of care [36,37].

Another ANTT practice that requires substantial improvement concerns the use of disinfectant agents. While most of the nurses performed skin antisepsis before attempting catheter insertion, the solution and technique used were not standardized, with nurses using different solutions, overlooking the drying time recommended by manufacturers, or using unsterile gauze. In several observations, nurses performed skin antisepsis, touched the puncture site again to confirm vein location, and proceeded with the procedure without performing skin antisepsis again. Similarly, during catheter maintenance, only 54% of the nurses scrubbed the needless connector before each use. Such practices undermine the quality and safety of the procedure, increasing the associated risk of skin contamination and bloodstream infection [16,35].

Flushing emerged in both study phases as a nursing practice that requires substantial improvement. Post-insertion flushing was performed only 67% of the time, with volumes varying between 2 mL and 10 mL of preservative-free 0.9% normal saline through bolus injection. Such compliance rate is below what is reported in other studies conducted in Portugal (53.3–84.2%) [34,38]. Likewise, catheter flushing before and after drug administration was performed 85% of the time, while catheter lock was performed 67%. Recent studies conducted in Portugal found that nurses often overlook catheter flushing due to high workloads and lack of recent training in vascular access, underestimating its importance [38,39]. In this study, nurses reported other factors that contribute to this reality, such as the shared use of intravenous bags to aspirate and prepare flushing solutions, increasing the risk of infection. Another factor was the regular omission of material for flushing, which can partially be explained by nurses’ knowledge gaps on flushing, storerooms being user-unfriendly, and the lack of an agreement between the ward nurses on where clinical material should be stored [40].

These results are concerning since international standards of care include statements on flushing as a gold standard practice that enhances catheter patency and function [41,42,43,44]. Flushing peripheral intravenous catheters before and after each administration of intravenous medication allows for the assessment of catheter patency, avoids the mixing of incompatible drugs and fluids, and reduces the build-up of fibrin and biofilm [16,45,46]. Future investment is required to promote standardized flushing practices between the ward nurses, as well as provide single-use materials that counteract the use of shared bags of normal saline, as reported in phase two. Such investment may reduce complications, such as phlebitis, infiltration, and PIVC-related bloodstream infection, which lead to multiple avoidable catheterizations during the admission period. International experts recommend the use of prefilled flush syringes, although the financial effect of its implementation across multiple clinical settings may influence their adoption [47].

In this study, catheter dressing emerged as another practice that was not standardized between the ward nurses, with some changing it daily as part of a care routine. Dressing disruptions are associated with an increased risk of skin contamination near the puncture site, leading to the migration of potentially pathogenic microorganisms through the inserted portion of the catheter, leading to the occurrence of PIVC-related bloodstream infections [48]. During phase two, several nurses reported recurrent episodes of loosened or damp dressings, which were not properly changed but reinforced with adhesive strips. This practice leads to the potential occlusion of the insertion site, undermining any subsequential efforts to monitor signs of local complications [16,47]. Although catheter dressing and securement practices remain an understudied area with mixed results [33,49], some studies have shown that dressing with reinforced borders decreases the risk of catheter accidental removal and dislodgement, and dressings can be used up to seven days if visibly clean and dry [33]. Such innovative materials may decrease care costs associated with routine dressing changes, avoid PIVC-related complications and repeated catheterizations, as well as improve patients’ experience.

Similarly, in phase one, most of the participants did not indicate the insertion date on the dressing, making it difficult for nurses in subsequent shifts to properly monitor the risk of complications. During the focus groups, several nurses reported that the lack of PIVC surveillance during each shift may explain the current complication rate. Remarkably, several nurses highlighted that nursing documentation does not reflect the actual maintenance care and surveillance performed by the ward nurses, which converges with previous international findings [50]. This may be explained by the underestimation of the risk associated with PIVCs by nurses [51] which, combined with the increasing complexity of care plans and poor nurse-patient ratios, leads to the omission of key practices that decrease the incidence of complications and catheter malfunctioning.

In this study, during phase one, a high complication rate (26%) was identified mainly due to infiltration and phlebitis, which are the two most reported complications internationally [52]. This finding is lower than most studies conducted in Portugal, with complications rates varying between 50% and 62.1% [33,34,39,53], although none of the studies was conducted in an oncology setting. Several risk factors can be attributed to the existing complication rate in this specific cohort, namely patient age, gender, immune system suppression or high body mass index [5,54,55]. However, nurses’ practices potentially explain some of the found complications, since poor patient involvement, adherence to aseptic technique, catheter stabilization and dressing integrity, and catheter flushing can lead to high incidence rates of infiltration and phlebitis.

This study must be analyzed considering its limitations. First, the study design and single study setting do not allow for the generalization of our findings, requiring a multicentric effort to present a comprehensive image of current PIVC-related practices and outcomes in oncology patients in Portugal. Second, this study does not account for the chemical properties of the intravenous medication and fluids administered during phase one, which may partially explain the found episodes of phlebitis and infiltration. Third, given that most peripheral intravenous catheters were inserted due to an impending surgery, we did not observe the practices performed by the nurses and anesthesiologists that cared for the study participants during the perioperative period and manipulated the devices.

Nonetheless, this study is the first conducted in Portugal that focuses on this specific cohort, which has been underrepresented in previous studies. This study contributes to the discussion of current PIVC-related practices in the country and, in combination with other research efforts, highlights the need for a national guideline on vascular access management (which is currently lacking [53,56], contrary to several European countries), nurses’ continuous training and education in this field, as well as the integration of standardized instruments and/or definitions to guide health professionals’ clinical decision [18,19,57]. Future interventions in Portuguese settings are required to standardize PIVC-related practices between healthcare professionals, contributing to the provision of evidence-informed and patient-centered care in this cohort.

## 5. Conclusions

This study was the first to report nurses’ practices in peripheral intravenous catheter insertion and maintenance in oncology patients in Portugal, using a mix-method approach to deepen the understanding of current challenges on a structural and procedural level. Our findings show that nurses’ insertion and maintenance practices are not standardized, leading to different approaches to vascular access care. The observed and reported practices diverge substantially from current international standards of care in specific areas, such as patient involvement, nurses’ adherence to the aseptic non-touch technique, catheter stabilization and dressing, and catheter flushing and locking. Future interventions are warranted to increase care quality and safety in this field.

## Figures and Tables

**Table 1:** Demographic and clinical characteristics of the study participants (*n* = 100).

Demographic and Clinical Variables	Patients’ Characteristics (*n* = 100)
Age	63.4 years (28–92; SD ± 14.2)
Sex	
Male	8%
Female	92%
Comorbidities	
Type 2 Diabetes Mellitus	16%
Arterial Hypertension	44%
Dyslipidemia	16%
Smoker	8%
Previous cancer treatment	
Chemotherapy	24%
Radiotherapy	2%
Hormone Therapy	2%
None	74%
Body Mass Index (kg/m^2^)	
Below 18.5	5%
18.5–24.9	26%
25.0–29.9	49%
30.0 and above	17%
Missing	3%
ENAV scale score	2.2 (1–5, SD ± 1.1)
Grade 1	32%
Grade 2	36%
Grade 3	21%
Grade 4	6%
Grade 5	5%
A-DM scale score	0.970 (0–5, SD ± 1.19)

SD = standard deviation; ENAV = Escala Nacional de Acessos Vasculares; A-DM = Escala A-DIVA Modificada.

**Table 2:** Nurses’ compliance with stipulated moments for hand hygiene (peripheral intravenous catheter insertion).

Moments for Hand Hygiene	Occurrences (%)
Before preparing the material needed for catheter insertion	42
After adjusting the patient environment	26
Before contact with the patient	100
After the procedure	100

**Table 3:** Nurses’ compliance with stipulated moments for hand hygiene (peripheral intravenous catheter maintenance).

Moments for Hand Hygiene	Occurrences (%)
Before preparing the material needed for catheter maintenance	55
After adjusting the patient environment	18
Before contact with the patient	98
After the procedure	100

## Data Availability

The data presented in this study are available on request from the corresponding author. The data are not publicly available due to ethical considerations, regarding personal information and to respect what was written in the signed informed consent.
